# CD39^+^PD-1^+^ regulatory T cells in melanoma: key drivers of systemic immunosuppression and prognostic biomarkers

**DOI:** 10.3389/fonc.2025.1724062

**Published:** 2025-12-05

**Authors:** Guanlin Qiao, Hongxia He, Xiaobing Wang

**Affiliations:** 1 The First Clinical Medical School, Shanxi Medical University, Taiyuan, Shanxi, China; 2 Department of Dermatology, The First Hospital of Shanxi Medical University, Taiyuan, Shanxi, China; 3 Department of Plastic and Reconstructive Surgery, First Hospital of Shanxi Medical University, Taiyuan, Shanxi, China

**Keywords:** melanoma, CD39+PD-1+ Treg, immunosuppression, prognosis, immunotherapy, biomarker

## Abstract

Melanoma remains a major challenge in oncology because of its aggressive behavior and intricate immune interactions. Advances in immunophenotyping and single-cell atlas technologies have revealed heterogeneous regulatory T cell (Treg) subsets, among which peripheral blood CD39^+^PD-1^+^ Tregs have emerged as key mediators of systemic immunosuppression. This review summarizes current evidence on their immunoregulatory functions, emphasizing their role in suppressing anti-tumor immunity and contributing to poor clinical outcomes. By integrating immune atlas data with clinical observations, we outline the mechanisms by which this subset shapes both the tumor microenvironment and systemic immune responses. We further discuss their potential as prognostic biomarkers and therapeutic targets to optimize immunotherapy strategies. In addition, we highlight how this subset interacts with other immunosuppressive pathways, reinforcing resistance to immune checkpoint inhibitors. Despite these advances, challenges remain in fully characterizing this population and translating findings into clinical application. This review provides a comprehensive overview of the significance of CD39^+^PD-1^+^ Tregs in melanoma immunopathology and highlights future directions to advance precision immunotherapy and improve patient prognosis.

## 1 Introduction

Melanoma is one of the most aggressive and life-threatening forms of skin cancer, originating from the malignant transformation of melanocytes. Despite advances in targeted and immune-based therapies, the prognosis for patients with advanced melanoma remains poor (1). A key barrier to effective treatment is the tumor’s ability to evade immune surveillance through multiple mechanisms within the tumor microenvironment (TME) that suppress anti-tumor immunity. Understanding these immune escape strategies is crucial for improving therapeutic efficacy and developing prognostic biomarkers.

Regulatory T cells (Tregs), a specialized CD4^+^ T cell subset characterized by FOXP3 and CD25 expression, are central mediators of immune tolerance. In melanoma, they exert potent immunosuppressive effects by inhibiting effector T cells and modulating other immune populations, and their accumulation is associated with poor prognosis and resistance to immune checkpoint inhibitors (ICIs). Recent single-cell RNA sequencing (scRNA-seq) studies have revealed the heterogeneity and plasticity of tumor-infiltrating Tregs, underscoring their diverse roles and therapeutic potential (1, 2).

CD39 functions far beyond its role on regulatory T cells and is increasingly recognized as a central modulator of the tumor microenvironment (TME). As an ectonucleotidase that hydrolyzes extracellular ATP and ADP into AMP, CD39 regulates purinergic signaling across tumor cells, endothelial cells, myeloid populations, and exhausted CD8^+^ T cells. Through the generation of adenosine and suppression of ATP-mediated inflammatory signaling, CD39 promotes the expansion of immunosuppressive cell subsets, facilitates VEGF-dependent angiogenesis, and supports metabolic programs—such as oxidative phosphorylation and lipid utilization—that enable tumor survival and progression. Recent evidence, including a 2024 study published in Cancer Lett, highlights CD39 as a key upstream regulator linking immune suppression, angiogenesis, and metabolic adaptation within the TME (3). These broader functions underscore the importance of contextualizing CD39 at the TME level before focusing on its specific role in PD-1^+^ regulatory T cells.

Recent studies have revealed that CD39/CD73-mediated adenosine metabolism and PD-1 checkpoint signaling converge to establish an immunosuppressive network across multiple tumor types, particularly melanoma. This dual axis suppresses effector T cell metabolism while stabilizing regulatory phenotypes, thereby shaping immune exclusion within the tumor microenvironment (4–7).

Mechanistically, CD39 hydrolyzes extracellular ATP (eATP) to AMP, which is subsequently converted to adenosine by CD73. The resulting adenosine-rich milieu suppresses effector T cell activation through A2A and A2B receptor signaling, while supporting Treg proliferation and functional stability. Concurrently, PD-1 engagement recruits the phosphatase SHP-2, which dephosphorylates CD3ζ and ZAP70, attenuating downstream TCR signaling and maintaining FOXP3 expression within Tregs. Together, these pathways form a coordinated metabolic–checkpoint regulatory circuit that reinforces immune suppression in melanoma (4–6).

Building upon these mechanisms, a distinct subset of CD39^+^PD-1^+^ regulatory T cells (Tregs) has been identified in both peripheral blood and melanoma tissues. This subset integrates adenosine metabolism with checkpoint signaling, conferring potent immunosuppressive capacity and correlating with unfavorable clinical outcomes. Functionally, CD39-generated adenosine inhibits effector T cell activation, while PD-1 signaling enhances Treg survival and suppressive function, collectively establishing a systemically immunosuppressive phenotype that contributes to tumor immune evasion (8, 9).

Single-cell immune profiling and transcriptomic analyses have characterized this subset as enriched in advanced melanoma and linked to immunosuppressive cytokine production, T cell exhaustion, and metabolic reprogramming (10, 11). CD39^+^PD-1^+^ Tregs express elevated levels of co-inhibitory receptors such as CTLA-4, TIGIT, and LAG-3, along with tissue residency markers, reflecting their specialized role in promoting an immune-excluded phenotype and resistance to ICIs (9, 12, 13).

Single-cell immune mapping has further highlighted the significance of this Treg subset as a key driver of systemic immunosuppression and poor prognosis (14, 15). Incorporating CD39 and PD-1 expression into prognostic gene signatures or immune-related risk scores has demonstrated strong predictive value for patient outcomes and immunotherapy response (16–19).

Collectively, these findings underscore the central role of peripheral blood CD39^+^PD-1^+^ Tregs in systemic immune suppression and melanoma progression. Integrating single-cell profiling with clinical data provides a foundation for novel therapeutic strategies targeting this subset to restore effective anti-tumor immunity and improve outcomes. This review summarizes their immunological characteristics, underlying mechanisms, and prognostic significance, providing a framework for future translational research (20).

**Figure 1:**
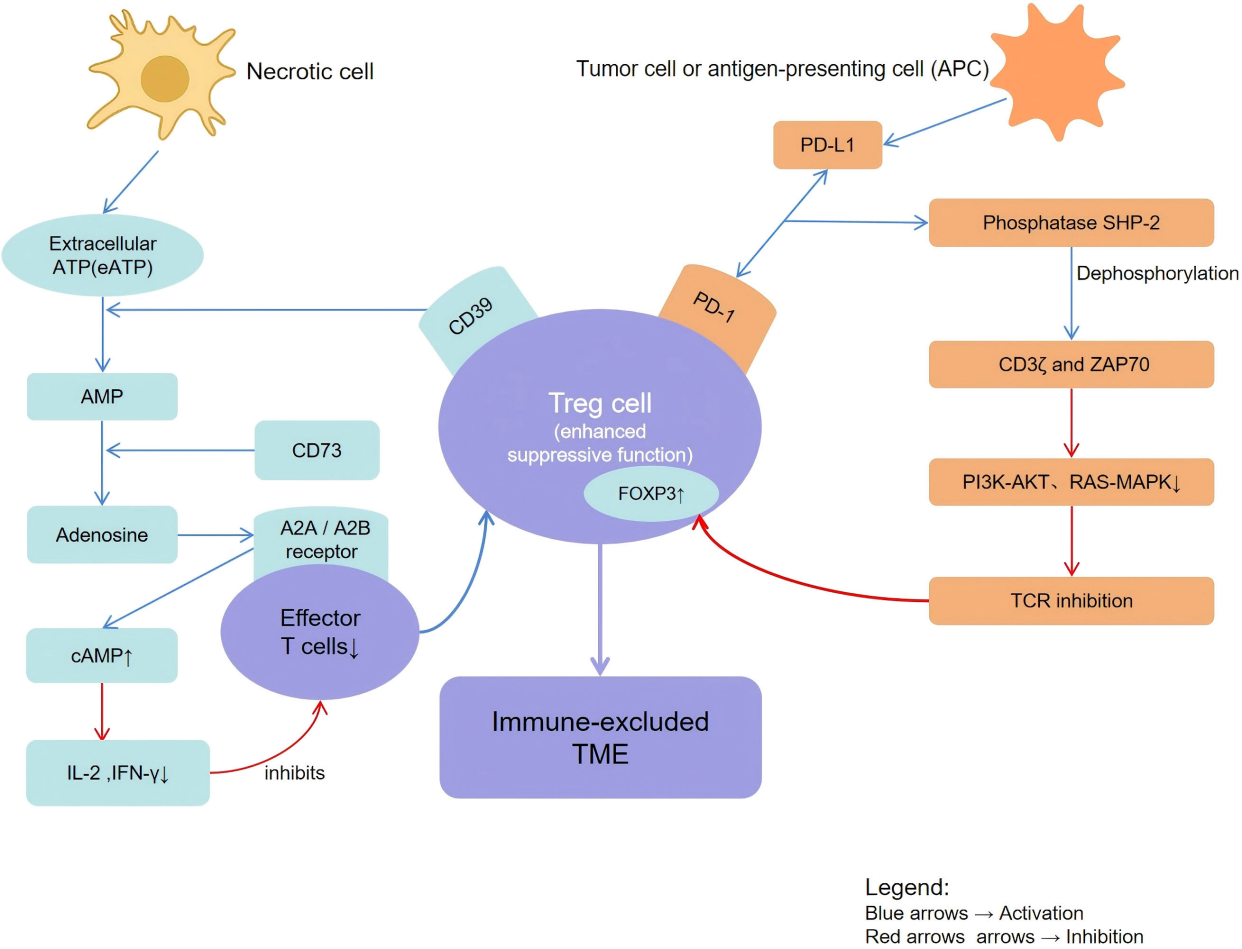
Schematic representation of CD39^+^PD-1^+^ regulatory T cell–mediated immunosuppressive mechanisms in the melanoma tumor microenvironment. Necrotic cells release extracellular ATP, which is hydrolyzed by CD39 into AMP and subsequently converted by CD73 into adenosine, leading to increased intracellular cAMP (↑cAMP) and immune suppression. Adenosine engages A2A/A2B receptors on effector T cells, dendritic cells, and macrophages to inhibit pro-inflammatory signaling. PD-1 signaling and SHP-2–mediated pathways attenuate TCR activation (TCR inhibition) and promote T-cell exhaustion. Arrows indicate stimulatory or inhibitory interactions, as specified in the pathway.

A conceptual schematic illustrating the convergence of the CD39/CD73–adenosine and PD-1–SHP2–TCR inhibitory pathways in regulatory T cells is shown in **Figure 1**.

## 2 Immunological and biological features of CD39^+^PD-1^+^ regulatory T cells in melanoma

### 2.1 Immunophenotypic characteristics of the CD39^+^PD-1^+^ Treg subpopulation

#### 2.1.1 Functional roles of CD39 and PD-1 in Treg-mediated immune regulation

CD39 and PD-1 are key regulators of immune homeostasis, particularly in the function of regulatory T cells (Tregs), which maintain tolerance and modulate immune responses in pathological contexts such as cancer. CD39, an ectonucleotidase, hydrolyzes extracellular ATP and ADP into AMP, subsequently converted into immunosuppressive adenosine by CD73 (21).

Extracellular ATP is largely released by necrotic tumor cells and metabolically stressed stromal or immune cells, where it functions as a prototypical danger-associated molecular pattern that triggers inflammatory signaling. CD39 catalyzes a stepwise breakdown of extracellular ATP and ADP into AMP, which is subsequently dephosphorylated by CD73 to generate adenosine within the tumor milieu. This cascade transforms inflammatory ATP signaling into immunosuppressive adenosine accumulation. By stimulating A2A and A2B adenosine receptors, adenosine increases intracellular cAMP and triggers PKA-dependent pathways, thereby suppressing IL-2 and IFN-γ secretion while enhancing the proliferation and stability of regulatory T cells. Upon PD-1 ligation, the phosphatase SHP-2 is recruited via ITIM/ITSM motifs, leading to dephosphorylation of CD3ζ and ZAP70 and consequent attenuation of downstream PI3K–AKT and RAS–MAPK signaling cascades. In Tregs, PD-1 signaling reinforces FOXP3 stability and oxidative metabolism, sustaining suppressive function (22, 23).

This pathway leads to adenosine accumulation, dampening effector T cell activity and supporting immune tolerance. High CD39 expression marks Treg subsets with enhanced suppressive capacity, contributing to tumor immune evasion (2, 24). In CAR-Treg models, CD39^+^ Tregs display reduced cytotoxicity but greater regulatory function (25).

PD-1, a classical immune checkpoint receptor expressed on T cells including Tregs, inhibits T cell activation through PD-L1/PD-L2 binding, maintaining peripheral tolerance. In Tregs, PD-1 expression reflects an activated or exhausted state, associated with enhanced suppressive capacity in cancer and other pathological settings (26–28).

Co-expression of CD39 and PD-1 identifies a potent immunosuppressive Treg subset enriched within tumor microenvironments. These cells strongly suppress local and systemic immunity. In ovarian cancer, CD39^+^PD-1^+^CD103^+^ Tregs exhibit an activated/exhausted tumor-resident phenotype associated with poor prognosis (29), and similar findings have been reported in papillary thyroid carcinoma (26). CD39-generated adenosine and PD-1 signaling synergize to inhibit effector T cell activity, fostering immune escape and adverse clinical outcomes (2, 30). Mechanistically, adenosine engages A2AR on effector T cells, while PD-1 signaling attenuates TCR pathways, jointly suppressing anti-tumor responses (31, 32). Aberrant CD39 and PD-1 expression is also implicated in autoimmune diseases (e.g., SLE) and infections (e.g., COVID-19, HIV) (33–35), indicating broader immunoregulatory roles.

In summary, co-expression of CD39 and PD-1 defines a highly suppressive Treg phenotype. Targeting this dual axis may restore anti-tumor immunity, and combination strategies involving CD39 inhibition and PD-1 blockade are under clinical investigation (2, 30).

#### 2.1.2 Single-cell profiling reveals a distinct immunosuppressive phenotype of CD39^+^PD-1^+^ Tregs

Single-cell RNA sequencing and multiparameter flow cytometry have revealed the transcriptional and phenotypic features of CD39^+^PD-1^+^ Tregs within tumors. This subpopulation displays a distinct immunosuppressive gene signature enriched for IL-10, TGF-β, and multiple checkpoint molecules, including CTLA-4 and TIGIT, consistent with a highly activated yet exhausted phenotype (29, 36). Flow cytometry confirms this surface marker co-expression, distinguishing these cells from other Treg subsets.

Single-cell datasets from melanoma lesions (e.g., GSE156326, GSE120575) reveal a PD-1^+^CD39^+^ Treg subpopulation characterized by enhanced fatty-acid uptake and oxidative phosphorylation (OXPHOS). CD39 facilitates lipid acquisition to fuel mitochondrial respiration, while PD-1 signaling stabilizes FOXP3 expression, resulting in a hyper-suppressive phenotype rather than functional exhaustion. These findings suggest that PD-1 single-positive Tregs may transition into PD-1^+^CD39^+^ Tregs under lipid-rich, hypoxic tumor conditions (6, 37, 38).

Metabolic profiling shows that CD39^+^PD-1^+^ Tregs rely heavily on oxidative phosphorylation and lipid metabolism to support survival and sustained suppression. This metabolic program contrasts with the glycolytic metabolism of effector T cells, suggesting an adaptive advantage and potential therapeutic vulnerability (29).

Spatial single-cell analyses further reveal preferential localization of these Tregs in immunosuppressive niches adjacent to exhausted CD8^+^ T cells, reinforcing local immune escape (39). Upregulation of residency markers such as CD103 supports their retention within tumor and peripheral compartments.

Spatial transcriptomic analyses reveal that CD39^+^PD-1^+^ Tregs are spatially enriched near exhausted CD8^+^ T cells and M2-polarized macrophages in hypoxic perivascular regions, collectively establishing adenosine-dominated immunosuppressive microdomains. This spatial coupling highlights how adenosinergic metabolism and checkpoint signaling cooperate to shape immune-excluded architectures in melanoma (40).

In summary, single-cell immune profiling highlights the CD39^+^PD-1^+^ Treg subset as a metabolically specialized and transcriptionally distinct population that sustains potent immunosuppression in cancer (29, 36, 39).

#### 2.1.3 Compartmental distribution of CD39^+^PD-1^+^ Tregs in peripheral blood and tumor tissue

The distribution of CD39^+^PD-1^+^ Tregs differs markedly between peripheral blood and tumor tissue, reflecting distinct roles in systemic and local immune regulation. Elevated frequencies in peripheral blood are observed in melanoma and other cancers, indicating systemic immunosuppression associated with poor outcomes (41). These cells produce adenosine and express PD-1, broadly dampening anti-tumor immunity. Longitudinal immune profiling has identified proliferating CD39^+^PD-1^+^ Tregs in peripheral blood as predictors of poor immunotherapy response and relapse (42).

Within the tumor microenvironment, these Tregs accumulate and contribute to immune escape. In papillary thyroid carcinoma and clear cell renal cell carcinoma, high CD39 and PD-1 expression correlates with recurrence and poor prognosis (26, 43). Their localized enrichment promotes an immunosuppressive niche through adenosine production and checkpoint signaling, inhibiting CD8^+^ T cell function.

This differential distribution reflects two immunological layers: a systemic regulatory network in peripheral blood and a localized immunosuppressive niche in tumors. Similar compartmentalization has been described in pancreatic ductal adenocarcinoma (44).

Overall, peripheral enrichment reflects systemic suppression, while intratumoral accumulation drives local immune escape. These dual roles should be considered when designing therapeutic strategies targeting CD39 and PD-1 pathways.

### 2.2 Mechanistic basis of CD39^+^PD-1^+^ Treg–mediated immune suppression in melanoma

#### 2.2.1 Adenosine signaling as a core immunosuppressive pathway

In melanoma, oncogenic BRAF activation drives the IL-6–STAT3 axis that enhances CD39 expression on tumor-infiltrating Tregs and macrophages. Concurrently, microphthalmia-associated transcription factor (MITF) regulates extracellular ATP release from melanoma cells, providing substrate for CD39 enzymatic activity and fueling adenosine generation. PD-1-mediated SHP-2 signaling cooperates with CD36-driven lipid metabolism, stabilizing FOXP3 and promoting oxidative phosphorylation. Together, these pathways establish a dual inhibitory loop reinforcing immune exclusion and checkpoint resistance within melanoma lesions (22, 23, 37, 38).

The adenosine-mediated immunosuppressive pathway plays a pivotal role in modulating immune responses within the tumor microenvironment, particularly through the enzymatic activity of CD39, which catalyzes the conversion of extracellular ATP to adenosine. CD39, an ectonucleotidase expressed on various immune and stromal cells, hydrolyzes pro-inflammatory ATP into AMP, which is subsequently converted to immunosuppressive adenosine by CD73. This enzymatic cascade leads to the accumulation of extracellular adenosine, a potent immunosuppressive metabolite that exerts its effects primarily via engagement of adenosine receptors, especially the A2A receptor (A2AR) on effector T cells and natural killer (NK) cells. Activation of A2AR triggers intracellular signaling pathways that suppress T cell proliferation, cytokine production, and cytotoxic functions, thereby dampening anti-tumor immunity and facilitating tumor immune evasion (45–48).

The immunosuppressive effects of adenosine signaling extend beyond T cells, influencing dendritic cells, macrophages, and myeloid-derived suppressor cells to promote an immune-tolerant tumor microenvironment (49, 50). Hypoxia in solid tumors upregulates CD39 and CD73 expression, enhancing adenosine production and reinforcing immunosuppression (51, 52). This hypoxia–adenosine axis not only inhibits effector immune cells but also promotes regulatory T cell (Treg) function and expansion, further contributing to immune tolerance (53, 54).

Therapeutic strategies targeting this pathway—including inhibitors of CD39/CD73 and antagonists of adenosine receptors—have shown promise in preclinical models by restoring T cell activity and enhancing responses to immune checkpoint blockade (55–57). Novel nanomedicine and biomaterial-based delivery systems are being explored to improve efficacy and specificity (45, 58). Collectively, the CD39–adenosine–A2AR axis represents a critical immunosuppressive mechanism and promising target for melanoma immunotherapy.

#### 2.2.2 PD-1 signaling and Treg functional regulation

The programmed cell death protein 1 (PD-1) signaling pathway plays a central role in T cell regulation, enhancing the immunosuppressive capacity of Tregs. PD-1 delivers inhibitory signals upon binding to PD-L1 or PD-L2, attenuating T cell receptor (TCR) signaling and downstream activation pathways. While this mechanism maintains peripheral tolerance, it also contributes to tumor immune evasion by promoting Treg-mediated suppression in the tumor microenvironment.

Mechanistically, PD-1 signaling recruits SHP-2 to ITIMs, dephosphorylating key signaling molecules downstream of TCR and CD28, thereby suppressing PI3K/Akt and ERK pathways and reducing effector T cell proliferation and cytokine production. Paradoxically, PD-1 engagement enhances Treg stability, survival, and suppressive function. Elevated PD-1 expression on Tregs correlates with increased immunosuppressive activity in cancer, autoimmune diseases, and chronic infections (59–61).

In ovarian and colorectal cancers, PD-1/PD-L1 signaling promotes Treg differentiation, recruitment, and TGF-β production, contributing to immune tolerance and poor prognosis (60, 62). PD-1 also influences Treg aging and cytokine profile shifts (e.g., IFN-γ → IL-10) (59), modulating immune balance.

PD-1 blockade, while reinvigorating effector T cells, can paradoxically expand tumor-infiltrating Tregs and induce hyperprogressive disease in some settings (63, 64). This is driven by increased IL-2 production from reactivated CD8^+^ T cells, which enhances ICOS expression and Treg survival. Moreover, PD-1 signaling intersects with metabolic and checkpoint pathways (e.g., mTOR-S6 axis, PI3K/Akt/ERK), affecting Treg metabolism and migration (65–67).

In summary, PD-1 signaling suppresses effector T cells and simultaneously amplifies Treg-mediated immunosuppression through enhanced differentiation, stability, and cytokine production. Understanding this dual role is essential for optimizing combination immunotherapies.

#### 2.2.3 Intercellular crosstalk and tumor microenvironment remodeling

**Figure 2:**
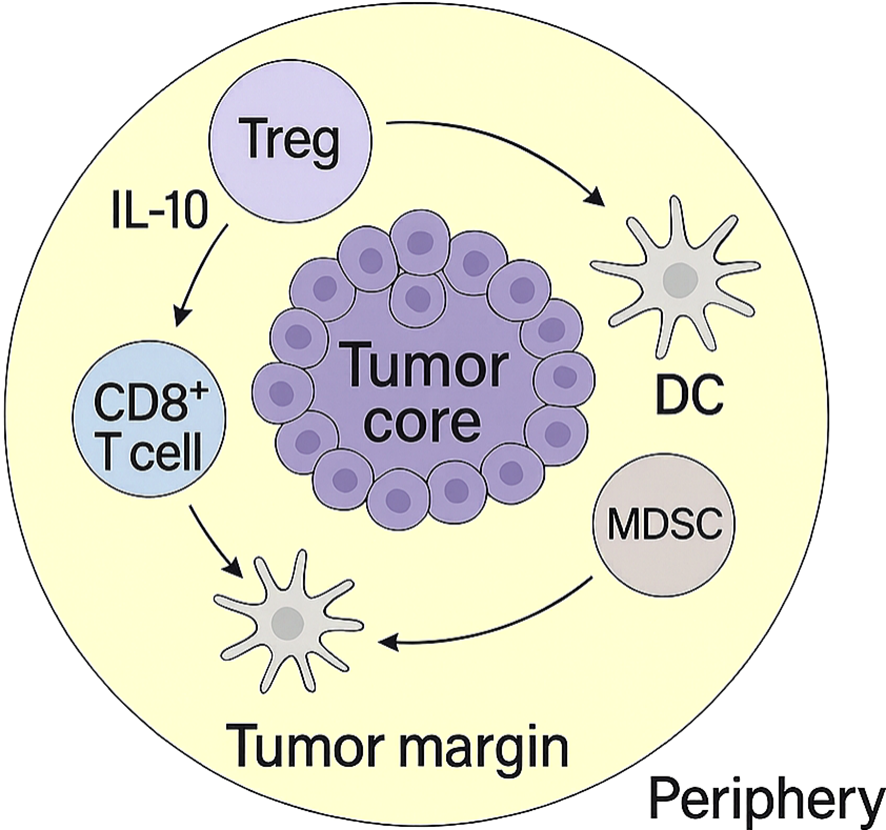
Spatial organization of immunosuppressive cells in the tumor microenvironment. CD39^+^PD-1^+^ Tregs, dendritic cells (DCs), MDSCs, and CD8^+^ T cells are distributed across the tumor core, margin, and periphery. This spatial structure underlies coordinated immune suppression and T cell exclusion.

CD39^+^PD-1^+^ Tregs orchestrate complex immunosuppressive networks within the tumor microenvironment (TME) through coordinated cytokine signaling, metabolic regulation, and contact-dependent interactions. These Tregs impair dendritic cell (DC) maturation by reducing IL-12 production and downregulating costimulatory molecules such as CD80 and CD86, promoting tolerogenic DC programs (68). They also release IL-10 and TGF-β, which further inhibit antigen presentation and reinforce DC dysfunction (69). In parallel, signaling through A2A/A2B adenosine receptors skews macrophages toward an M2-like phenotype characterized by ARG1, CD206, and IL-10 expression, contributing to stromal remodeling, angiogenesis, and enhanced immune suppression (42, 70) **Figure 2**.

Spatial profiling studies reveal that CD39^+^PD-1^+^ Tregs frequently colocalize with exhausted CD8^+^ T cells in hypoxic, adenosine-rich niches, where sustained PD-1–SHP-2 signaling dampens TCR activation and restricts cytotoxic effector function (39, 71). High infiltration of this subset correlates with poor prognosis and resistance to checkpoint blockade therapy in melanoma and other solid tumors (72, 73). These Tregs also modulate TME metabolism—for example, by enhancing PGE2 production through COX-2 signaling—which strengthens their suppressive activity and further limits antitumor immunity (74, 75). Collectively, these layers of cytokine- and metabolism-driven regulation position CD39^+^PD-1^+^ Tregs as central organizers of immunoregulatory networks in melanoma (36).

Beyond these interactions, CD39^+^PD-1^+^ Tregs engage in reciprocal crosstalk with additional immunosuppressive populations that further consolidate the tolerogenic TME. First, bidirectional communication with myeloid-derived suppressor cells (MDSCs) reinforces metabolic suppression: adenosine generated by Treg-expressed CD39/CD73 expands both monocytic and granulocytic MDSCs, while MDSCs release ATP and immunomodulatory metabolites that enhance CD39 expression and stabilize the suppressive Treg phenotype (75, 76). Second, tumor-associated macrophages (TAMs) not only undergo M2 polarization under adenosine and TGF-β signaling but also supply extracellular ATP and CD39^+^/CD73^+^ vesicles, amplifying the adenosinergic circuit and fostering mutual reinforcement of Treg and TAM regulatory programs (70, 77). Third, extracellular vesicles (EVs) enriched in CD39, CD73, or ATP/AMP act as mobile metabolic platforms that enhance adenosine production and mediate long-range immunosuppression, integrating tumor cells, macrophages, and Tregs into a coordinated metabolic network (58, 77).

Together, these multidimensional interactions complete the immunosuppressive framework driven by CD39^+^PD-1^+^ Tregs, providing a mechanistic basis for their role in shaping TME composition, driving immune evasion, and contributing to immunotherapy resistance in melanoma. The key immunoregulatory mechanisms are summarized in **Table 1**.

**Table 1:** Key immunoregulatory mechanisms of CD39^+^PD-1^+^ regulatory T cells in the tumor microenvironment.

Mechanism category	Key pathway/Molecular events	Affected immune cells	Functional outcome	Representative references
Adenosinergic pathway (CD39–CD73 axis)	CD39 converts eATP → AMP; CD73 converts AMP → adenosine; adenosine binds A2A/A2B receptors	Dendritic cells (DCs), CD8^+^ T cells, macrophages	Inhibits DC maturation; reduces IL-12; suppresses CD8^+^ cytotoxicity; promotes M2 macrophage polarization	(8, 36, 39, 68, 70)
PD-1/SHP-2 signaling	PD-1 engagement recruits SHP-2 → dephosphorylates CD3ζ/ZAP70 → inhibits TCR activation	CD8^+^ T cells, Tregs	Induces T-cell exhaustion; suppresses TCR signaling	(39, 60)
cAMP-mediated metabolic suppression	Adenosine increases intracellular cAMP → inhibits effector signaling	CD8^+^ T cells, NK cells	Reduces cytokine production and cytotoxicity	(32, 45)
Cytokine-mediated suppression	IL-10, TGF-β secretion by CD39^+^PD-1^+^ Tregs	DCs, CD8^+^ T cells, macrophages	Promotes tolerogenic DCs and suppresses effector responses	(8, 70)
Cell–cell contact-dependent suppression	CTLA-4, PD-1, and other checkpoint interactions	APCs, T cells	Downregulates costimulatory molecules (e.g., CD80/CD86) and limits T-cell priming	(24, 55)
Microenvironment remodeling	Tregs colocalize with exhausted CD8^+^ T cells in hypoxic/adenosine-rich niches	Multiple immune subsets	Creates immune-excluded TME, enhances suppression	(39, 71)
Treg–MDSC metabolic reciprocity (NEW)	Treg-derived adenosine expands monocytic & granulocytic MDSCs; MDSCs release ATP/ROS to upregulate CD39 and stabilize suppressive Treg phenotype	MDSCs, Tregs	Reciprocal amplification of metabolic suppression; CD8^+^ T-cell inhibition	(75, 76)
Bidirectional adenosine regulation with TAMs (NEW)	Adenosine induces M2 polarization; TAM-derived ATP and CD39/CD73^+^ vesicles reinforce adenosine cycle	Macrophages (TAMs), Tregs	M2 polarization, angiogenesis, increased immunosuppression	(70, 77)
Extracellular vesicle (EV)-mediated CD39/CD73 regulation (NEW)	Tumor-derived EVs transport CD39/CD73 or ATP/AMP; EV-associated molecules suppress DC activation and expand Tregs	DCs, Tregs, macrophages	Impaired antigen presentation; reinforcement of Treg-driven suppression	(58, 77)

**Table 2:** Therapeutic strategies targeting CD39^+^PD-1^+^ regulatory T cells and the adenosinergic immunosuppressive axis.

Therapeutic category	Strategy/agents	Mechanism of action	Current evidence/stage	Representative references
CD39 inhibition	Small-molecule inhibitors; CD39-blocking antibodies	Block ATP → AMP hydrolysis; reduce extracellular adenosine; restore effector T-cell activity	Preclinical models; early-phase translational research	(56, 57, 72)
CD73 inhibition	AB680 (Quemliclustat); monoclonal antibodies	Block AMP → adenosine conversion; enhance anti-tumor immunity	Phase I/II clinical trials	(55, 56)
A2A/A2B receptor antagonists	DZD2269; M1069; other A2A/A2B blockers	Prevent adenosine signaling through A2A/A2B; reverse T-cell suppression; reduce M2 macrophage polarization	Multiple agents in clinical and preclinical pipelines	(47, 49, 50)
Combined adenosine-pathway targeting + PD-1 blockade	Anti–PD-1 + CD39/CD73/A2A inhibitors	Synergistic reactivation of exhausted CD8^+^ T cells; reduced Treg-mediated suppression	Strong preclinical evidence; emerging clinical evaluation	(60, 76)
Treg-modulating therapeutics	TGF-β blockade; CTLA-4 modulation	Reduce Treg differentiation/trafficking; weaken Treg suppressive function	Preclinical and translational studies	(24, 80, 85)
Nanomedicine/advanced delivery platforms	Biomimetic nanoparticles; ultrasound-responsive systems	Target adenosine axis; enhance drug delivery; reshape TME	Cutting-edge preclinical research	(51, 58)

**Table 3:** Technological platforms enabling high-resolution profiling of CD39^+^PD-1^+^ regulatory T cells in the tumor microenvironment.

Technology	Key features	What it reveals about CD39^+^PD-1^+^ Tregs	Strengths	Limitations	Representative references
Single-cell RNA sequencing (scRNA-seq)	Transcriptome-wide single-cell profiling	Identifies Treg clusters; defines exhaustion, activation, metabolic programs	High resolution; unbiased discovery	Limited protein information; requires validation	(14, 39, 42)
CyTOF/high-dimensional flow cytometry	Multiparameter protein-level immune profiling	Defines Treg phenotypes (FOXP3^+^, HLA-DR^+^, CD38^+^, CD39^+^, PD-1^+^ subsets)	High protein resolution; robust surface markers	Requires predefined markers	(26, 42, 84)
ATAC-seq/single-cell chromatin profiling	Maps chromatin accessibility	Reveals epigenetic states driving suppressive programs and exhaustion	Links transcription factors to function	Lower throughput; complex analysis	(12, 13)
Spatial transcriptomics	Spatial transcriptome mapping within TME	Shows localization of CD39^+^PD-1^+^ Tregs; co-localization with exhausted CD8^+^ T cells	Adds spatial context to function	Lower resolution than scRNA-seq	(14, 40)
Proteomics/metabolomics	Quantifies proteins & metabolic signatures	Illuminates adenosine pathway activity (CD39/CD73/ADA), metabolic suppression	Functional insight	Requires bulk tissue in many cases	(23, 32, 45)

### 2.3 Prognostic implications of the CD39^+^PD-1^+^ Treg subpopulation in melanoma

The immune architecture of melanoma is profoundly shaped by the balance between effector and regulatory T cell populations, with CD39^+^PD-1^+^ T cell subsets emerging as key immunological determinants of clinical outcomes. Elevated frequencies of CD39^+^PD-1^+^ regulatory T cells (Tregs) in both peripheral blood and tumor tissues reflect a systemic immunosuppressive state and are strongly associated with disease progression, recurrence, and reduced overall survival (78, 79).

Previous studies have shown that the observations specifically derive from primary uveal melanoma cohorts (80). Comparable high-resolution analyses in cutaneous and acral melanoma remain limited, underscoring the need for subtype-specific profiling to clarify prognostic heterogeneity across melanoma lineages (78).

The co-expression of CD39 and PD-1 endows these Tregs with potent suppressive capacity through adenosine-mediated and PD-1–dependent inhibitory pathways, promoting immune evasion, tumor progression, and resistance to immune checkpoint blockade (78). In uveal melanoma, particularly in tumors with monosomy 3, increased infiltration of CD39^+^PD-1^+^ Tregs and exhausted CD8^+^ T cells correlates with enhanced immune suppression and poor clinical outcomes. Their detectability through minimally invasive techniques, such as flow cytometry and liquid biopsy, underscores their potential as circulating prognostic and predictive biomarkers as well as therapeutic targets.

Recent mechanistic studies have further shown that PD-1 signaling functions as a critical negative regulator of Treg activity. PD-1 deficiency or blockade leads to compensatory upregulation of co-inhibitory receptors—such as CD30, CTLA4, and GITR—through enhanced STAT5 signaling, generating a hyperfunctional Treg phenotype that amplifies immunosuppression and fosters an immune-cold tumor microenvironment (37). This pattern mirrors the biological behavior of CD39^+^PD-1^+^ Tregs, reinforcing their role as a negative prognostic factor and a potential mediator of therapeutic resistance.

In parallel, CD39^+^PD-1^+^ effector T cells—especially CD39^+^CD103^+^PD-1^+^ tissue-resident memory (Trm) CD8^+^ T cells—have been identified as favorable prognostic indicators. In a large cohort of stage III melanoma patients receiving adjuvant anti–PD-1 therapy, higher intratumoral frequencies of this subset were significantly associated with prolonged recurrence-free survival (1-year RFS: 78.1% vs. 49.9%; HR 0.32, 95% CI 0.15–0.69; p = 0.0036) (81, 82). Their preferential localization in close proximity to melanoma cells reflects a tumor-reactive phenotype with robust cytotoxic potential. Incorporating the abundance of CD39^+^PD-1^+^ T cells into multivariable prognostic models substantially improves recurrence risk stratification.

Importantly, these two CD39^+^PD-1^+^ populations exhibit opposite biological and clinical implications: Trm CD8^+^ T cells signify a favorable immune contexture and improved prognosis, whereas CD39^+^PD-1^+^ Tregs represent a dominant immunosuppressive axis linked to poor outcomes and therapeutic resistance (37, 78, 80). This immunological duality highlights the prognostic and predictive significance of CD39^+^PD-1^+^ T cell subsets in melanoma and supports their integration into precision immuno-oncology strategies for patient stratification, disease monitoring, and the rational design of combination therapies.

### 2.4 Therapeutic roles and clinical application prospects of CD39^+^PD-1^+^ Tregs in immunotherapy

#### 2.4.1 Effects of immune checkpoint inhibitors on CD39^+^PD-1^+^ Tregs

Immune checkpoint inhibitors (ICIs), particularly PD-1 blockade, have transformed melanoma therapy by reinvigorating exhausted T cells. However, their impact on immunosuppressive Treg subsets—such as peripheral CD39^+^PD-1^+^ Tregs—remains complex and often incompletely addressed by monotherapy (83). PD-1 inhibitors can partly relieve suppression by disrupting PD-1–mediated inhibitory signaling, thereby enhancing effector T-cell activity. Yet CD39, highly expressed on this subset, continuously hydrolyzes extracellular ATP to AMP (converted to adenosine by CD73), sustaining an adenosine-rich, immunosuppressive milieu that persists despite PD-1 blockade. Thus, the CD39–adenosine axis functions as a parallel checkpoint that limits ICI efficacy.

Even under PD-1 blockade, CD39-mediated adenosine accumulation can maintain an immunosuppressive milieu, limiting therapeutic efficacy. Dual targeting of PD-1 and the adenosinergic pathway has shown synergistic activation of effector T cells and reversal of immune exhaustion in preclinical melanoma models (23, 37, 86).

Preclinical data support combination approaches. In murine epithelial ovarian cancer, pairing PD-1 blockade with agents targeting immunometabolic pathways (e.g., tryptophan metabolism) reduced immunosuppressive CD39^+^PD-1^+^ cells and improved antitumor immunity (87). In melanoma and pancreatic cancer models, CD39^+^ Treg abundance correlated with diminished responses to PD-1 monotherapy, implicating adenosine production as a resistance mechanism (37). Dual blockade strategies targeting PD-1 and CD39 synergistically restore proliferation and effector functions by reversing PD-1–driven exhaustion and preventing adenosine accumulation. Related multimodal regimens—such as PD-1 plus TIGIT inhibition in pancreatic ductal adenocarcinoma—further enhance T-cell activation and cytokine production (81). Photodynamic therapy combined with PD-1/PD-L1 blockade can also decrease CD39^+^ Tregs and augment cytotoxic CD8^+^ T cells, reinforcing the rationale for co-targeting adenosinergic suppression (89).

Collectively, while PD-1 blockade partially mitigates CD39^+^PD-1^+^ Treg–mediated suppression, the adenosinergic barrier remains critical. Integrated strategies that concurrently inhibit PD-1 and CD39 are promising for boosting ICI efficacy and improving outcomes in melanoma and other CD39^+^PD-1^+^ Treg–high malignancies.

#### 2.4.2 Therapeutic strategies targeting CD39^+^PD-1^+^ Treg subsets

Directly targeting the CD39^+^PD-1^+^ Treg compartment offers a route to overcome systemic immunosuppression and improve prognosis. One approach is the development of monoclonal antibodies or small-molecule inhibitors to deplete or functionally inhibit this subset. Inhibiting CD39 enzymatic activity reduces adenosine accumulation, restoring pro-inflammatory ATP signaling and antitumor immunity. In bladder cancer models, CD39 inhibition increased intratumoral NK cells, dendritic cells, and CD8^+^ T cells while decreasing Tregs, limiting tumor growth and prolonging survival (72). Given the association between elevated CD39^+^ Tregs and poor responses to PD-1 therapy (16), CD39 blockade may sensitize tumors to ICIs.

Because PD-1 expression marks an activated, suppressive Treg phenotype, PD-1/PD-L1 antibodies can modulate Treg function but may paradoxically expand PD-1^+^/CD39^+^ Tregs in some settings, underscoring the need for combination regimens (e.g., CD39 + PD-1 co-blockade) (42). Preclinical studies show synergistic antitumor effects with enhanced T-cell activation when combining these agents (2).

Adjunctive modalities can further potentiate efficacy. Photodynamic therapy (e.g., Chlorin e6–based PDT) reduces CD39^+^ Tregs and complements PD-1/PD-L1 blockade, amplifying abscopal immune effects in melanoma models (37). Small molecules that reprogram immunometabolism (e.g., amitriptyline affecting tryptophan metabolism) decrease CD39^+^PD-1^+^ T cells and synergize with PD-1 inhibitors in ovarian cancer (87).

Finally, engineered Treg approaches illustrate how tuning CD39 can modulate suppressive profiles: CAR-Tregs engineered with CD39 show reduced cytotoxicity and refined regulatory activity, suggesting avenues to fine-tune Treg biology for therapeutic ends—either to prevent autoimmunity or to counter tumor-promoting Tregs (25).

In summary, strategies include CD39 enzymatic inhibition, PD-1/PD-L1 blockade (preferably in rational combinations), and immunometabolic or modality-based adjuncts. Ongoing clinical translation of CD39 inhibitors and combination regimens may help dismantle the adenosinergic axis and restore effective immune surveillance (2, 88).

#### 2.4.3 Clinical application prospects of targeting CD39^+^PD-1^+^ Tregs

Therapeutically, combination strategies informed by mechanistic insights—such as PD-1 plus CD39/CD73 blockade or photodynamic therapy (PDT) combined with PD-1/PD-L1 inhibitors—hold promise for selectively depleting or reprogramming suppressive CD39^+^PD-1^+^ Tregs and enhancing abscopal immunity in melanoma models (89). These approaches provide a rationale for incorporating adenosinergic/Treg-targeted agents into next-generation immunotherapy combinations and for early-phase clinical trials stratifying patients by CD39/CD73 expression or adenosine-related signatures. See **Table 2** for details.

### 2.5 Research challenges and future prospects

#### 2.5.1 Limitations of existing studies

Current research on peripheral blood CD39^+^PD-1^+^ regulatory T cell (Treg) subsets in melanoma is constrained by several methodological and clinical limitations. A major issue is the limited sample size and absence of large-scale, multicenter validation. Most available studies rely on small cohorts or preclinical models, which reduces generalizability and statistical power. For example, investigations of T cell infiltrates in uveal melanoma revealed CD39^+^PD-1^+^CD8^+^ T-cell enrichment in high-risk M3 tumors (78), but these findings were based on localized tumor samples and have not been extensively validated across melanoma subtypes or diverse patient populations. This is critical given the immunological heterogeneity between cutaneous and uveal melanoma, as well as interpatient variability in immune responses.

Another limitation stems from mechanistic studies predominantly relying on *in vitro* or animal models, which cannot fully recapitulate the complexity of human immune responses. For instance, Ce6-based photodynamic therapy (PDT) has shown immunomodulatory effects in mouse models by inhibiting PD-1/PD-L1 and reducing CD39^+^ Tregs (89), but translation to clinical settings remains uncertain. Human immune regulation involves far greater heterogeneity and dynamic interactions between circulating and tumor-infiltrating immune cells.

In summary, the small scale of patient cohorts and heavy reliance on preclinical systems hinder the clinical translation of these findings. Future work should prioritize multicenter clinical studies and integrated approaches combining patient-derived samples, advanced single-cell techniques, and functional assays to bridge preclinical discoveries with clinical implementation.

#### 2.5.2 Technological and methodological advances driving deeper research

Recent advances in single-cell and spatial omics have transformed the study of CD39^+^PD-1^+^ Tregs. Single-cell RNA sequencing and CITE-seq have enabled high-resolution profiling of Treg subsets, revealing activation states, clonality, and functional heterogeneity in multiple cancers (4, 29, 90). These methods highlight that CD39^+^PD-1^+^ Tregs often represent terminally differentiated, highly suppressive populations that migrate from the periphery into tumor sites.

Multidimensional flow cytometry and CyTOF allow precise quantification of markers such as CD39, PD-1, Foxp3, and CD103, enabling dynamic monitoring of these Tregs during disease progression and treatment (16, 26). In breast cancer, elevated baseline CD39^+^ Tregs correlated with poor anti-PD-1 therapy response (16). Multiplex immunohistochemistry and seven-color immunofluorescence provide spatial localization data, showing close proximity between CD39^+^PD-1^+^ Tregs and exhausted CD8^+^ T cells in renal cell carcinoma (39), revealing their role in local immune suppression.

The integration of spatial transcriptomics and proteomics with single-cell data has uncovered preferential accumulation of CD39^+^PD-1^+^ Tregs in tumor centers or invasive margins, where they dampen effector responses (29, 39). Longitudinal profiling before and after therapy further exposes population dynamics and resistance mechanisms (42). Advanced bioinformatics and machine learning approaches integrate these datasets into prognostic models, improving patient stratification and identifying co-expressed targets such as TIGIT (29, 91).

In short, these technological advances have significantly deepened understanding of the phenotype, localization, and clinical relevance of CD39^+^PD-1^+^ Tregs, laying the foundation for future translational strategies.

Future progress hinges on understanding the temporal dynamics of CD39^+^PD-1^+^ Tregs across treatment. Longitudinal profiling shows these populations evolve with therapy: in anti-PD-1–treated HER2-negative breast cancer, non-responders had higher baseline CD39^+^ Tregs that further expanded post-treatment, implicating them in resistance (16). In nasopharyngeal carcinoma, proliferative Ki67^+^ CD39^+^PD-1^+^ Tregs increased during therapy among eventual relapsers, correlating with poor outcomes and highlighting their value as predictive biomarkers (42). Routine longitudinal monitoring could enable early resistance prediction and adaptive treatment.

Advances in single-cell and multi-omics (scRNA-seq, CyTOF, ATAC-seq, proteomics) now resolve Treg heterogeneity and functional states with high fidelity, identifying marker combinations (FOXP3, CD38, HLA-DR, CD39, PD-1) linked to recurrence or adverse prognosis (26, 42). Integrative multi-omics can map signaling circuits and metabolic programs, surfacing druggable nodes. Mechanistically, single-cell analyses continue to clarify how adenosine generation (CD39/CD73) and PD-1 signaling converge to suppress effector responses (76).

Together, these tools provide a foundation for biomarker-guided immunotherapy, enabling personalized treatment selection, adaptive ICI combinations, and resistance monitoring in future clinical practice (92–94). Details are shown in **Table 3**.

#### 2.5.3 Clinical prospects and challenges

Translating the biology of peripheral CD39^+^PD-1^+^ regulatory T cells (Tregs) into clinically actionable strategies presents both significant opportunities and substantial challenges. A major barrier remains the lack of methodological standardization. Heterogeneity in flow cytometry panels, gating strategies, and transcriptional or metabolic assays currently limits reproducibility across clinical studies. Establishing harmonized analytical frameworks will be essential for accurate quantification of CD39^+^PD-1^+^ Tregs and for integrating their profiles into prognostic tools, therapeutic monitoring, and biomarker-driven clinical trial design.

Meaningful clinical translation will also require closer collaboration across immunology, oncology, computational biology, and clinical sciences. Such interdisciplinary integration is crucial for advancing precision immune profiling and for identifying therapeutic windows in which modulation of CD39^+^PD-1^+^ Tregs improves antitumor immunity without precipitating excessive immune-related toxicity.

Another practical challenge involves balancing immune activation with immune tolerance. While immune checkpoint inhibitors—such as PD-1/PD-L1 blockade—enhance effector responses, they also increase the risk of autoimmune toxicity. CD39^+^PD-1^+^ Tregs, positioned at the interface of immunosuppression and immune activation, may serve as both biomarkers and therapeutic modulators. Precision modulation—rather than broad depletion—is therefore critical. Photodynamic therapy (PDT), for instance, can synergize with PD-1 blockade, reducing CD39^+^ Treg abundance and augmenting CD8^+^ T-cell activity, though toxicity monitoring remains essential (37).

Finally, differences among melanoma subtypes (e.g., cutaneous vs. uveal) demand tailored immunotherapeutic strategies. Uveal melanoma, which is characterized by enrichment of CD39^+^PD-1^+^ T cells, may require therapeutic approaches that integrate microenvironmental cues with systemic immune dynamics (80).

Recent advances in immunotherapy have highlighted several promising strategies targeting CD39^+^PD-1^+^ Tregs and the broader adenosinergic network. Selective CD73 inhibitors, including the small-molecule inhibitor AB680 (quemliclustat), have shown encouraging antitumor activity and are currently evaluated in combination with PD-1/PD-L1 blockade (56, 57). Furthermore, dual-target adenosine-axis strategies—such as CD39/CD73 bispecific antibodies and multitarget metabolic inhibitors—aim to overcome compensatory enzymatic redundancy and more effectively suppress adenosine production within the tumor microenvironment (TME) (3, 7, 55).

In parallel, extracellular vesicle (EV)–directed interventions are emerging as an innovative therapeutic approach. Tumor-derived EVs enriched in CD39, CD73, or ATP/AMP function as mobile metabolic platforms that propagate long-range adenosine-mediated immunosuppression. Strategies that inhibit EV biogenesis, block EV-associated ectonucleotidase activity, or prevent EV-mediated intercellular transfer may significantly attenuate Treg-dominated immune suppression and enhance cytotoxic lymphocyte responses (58, 77).

Another rapidly expanding therapeutic avenue involves Treg reprogramming and destabilization strategies. Modified IL-2 molecules—such as IL-2 muteins—and selective low-dose IL-2 regimens are being developed to preferentially expand effector lymphocytes while limiting Treg proliferation (30). Additional approaches seek to destabilize the suppressive Treg lineage by targeting transcriptional and metabolic pathways, including STAT3 inhibition, COX-2/PGE2 blockade, and disruption of the PD-1–SHP-2 axis, each of which can reduce Treg-mediated suppression in tumor tissues (61, 83, 85). Rather than systemic Treg depletion, these localized reprogramming strategies aim to maintain peripheral tolerance while alleviating intratumoral immune suppression.

Collectively, these emerging therapeutic modalities—including CD73 inhibition, dual adenosinergic targeting, EV-based interventions, and Treg reprogramming—offer promising avenues to refine melanoma immunotherapy. Integrating these approaches with robust biomarker systems and standardized immune monitoring may ultimately enable precise, context-specific modulation of CD39^+^PD-1^+^ Tregs to enhance clinical outcomes while minimizing toxicity.

## 3 Conclusion

The identification of the CD39^+^PD-1^+^ regulatory T cell (Treg) subset as a central driver of systemic immunosuppression in melanoma represents a pivotal advancement in tumor immunology. This subpopulation displays unique molecular signatures and potent suppressive activity that shape both the tumor microenvironment and systemic immune responses. The strong correlation between the abundance of peripheral CD39^+^PD-1^+^ Tregs and poor patient prognosis highlights their dual value as prognostic biomarkers and therapeutic targets.

While Tregs have long been implicated in tumor immune evasion, delineating this specific subset refines the immunological paradigm by emphasizing its distinct functional and metabolic features. This knowledge bridges clinical observations and mechanistic insights, enabling the development of more precise and rational immunotherapeutic strategies. In particular, integrating interventions that modulate CD39^+^PD-1^+^ Tregs with immune checkpoint inhibitors or metabolic reprogramming therapies holds promise for overcoming resistance and enhancing antitumor efficacy.

Nonetheless, significant challenges remain. Technical limitations in standardized detection and isolation, incomplete mechanistic understanding of their suppressive pathways, and uncertainties surrounding optimal therapeutic targeting strategies pose hurdles to clinical translation. Addressing these gaps will require advanced single-cell and spatial technologies, robust clinical validation, and carefully designed combinatorial regimens that modulate Treg activity without disrupting systemic immune balance.

Future investigations integrating single-cell multi-omics and spatial transcriptomics are essential to map the dynamic niches of CD39^+^PD-1^+^ Tregs and to identify combinatorial targets within the adenosinergic–checkpoint axis. Such approaches will refine prognostic evaluation and guide rational design of next-generation immunotherapies for melanoma (6, 40).

In summary, focusing on CD39^+^PD-1^+^ Tregs offers a transformative opportunity to personalize and intensify melanoma immunotherapy. Achieving this goal will depend on sustained interdisciplinary collaboration, large-scale clinical studies, and methodologically rigorous research to fully harness the therapeutic potential of this immunoregulatory subset—ultimately improving patient survival and advancing the frontier of precision oncology.
